# Breakthrough Hemolysis Associated With COVID-19 Vaccination and Active COVID-19 Infection in a Patient With Paroxysmal Nocturnal Hemoglobinuria Maintained on Pegcetacoplan: A Case Report

**DOI:** 10.7759/cureus.36240

**Published:** 2023-03-16

**Authors:** Mitchell C Boshkos, Kaila R Fives, Davong D Phrathep, Kevin D Healey, Miten Patel

**Affiliations:** 1 Internal Medicine, Lake Erie College of Osteopathic Medicine, Bradenton, USA; 2 College of Medicine, Lake Erie College of Osteopathic Medicine, Bradenton, USA; 3 College of Osteopathic Medicine, Lake Erie College of Osteopathic Medicine, Bradenton, USA; 4 Hematology and Medical Oncology, Cancer Specialists of North Florida, Jacksonville, USA

**Keywords:** breakthrough hemolysis, pegcetacoplan, covid-19, covid-19 vaccine, paroxysmal nocturnal hemoglobinuria (pnh)

## Abstract

Breakthrough hemolysis (BTH) is the return of hemolytic disease resulting in an overall increase in complement activation in a patient being treated for paroxysmal nocturnal hemoglobinuria (PNH) with complement inhibitors (CI). BTH after COVID-19 vaccination has only been reported in PNH patients treated with the traditional C5 CI eculizumab and ravulizumab. We report on a new association of BTH in a newly COVID-19 vaccinated, previously stable PNH patient treated with pegcetacoplan, a C3 CI.

The patient is a 29-year-old female diagnosed with PNH in 2017 and was started on eculizumab but was switched to pegcetacoplan in 2021 after continuing to exhibit symptomatic hemolysis. Subsequently, the patient returned to PNH remission serologically and symptomatically until her first COVID-19 vaccination. Since then, her lactate dehydrogenase (LDH) and hemoglobin counts have not fully returned to previous baseline levels, with significant exacerbations after her second COVID-19 vaccine and de novo COVID-19 infection. As of May 2022, the patient requires packed red blood cell transfusions every two to three months and has undergone a bone marrow transplant evaluation.

This case study suggests that the administration of the upstream C3 CI, pegcetacoplan, is associated with active extravascular hemolysis in the setting of COVID-19 vaccinations and active COVID-19 infection. The pathophysiology of this hemolysis is unclear as hemolysis could be related to the underlying complement factor deficiency or amplification of complement factors causing extravascular hemolysis. There are conflicting reports in the literature regarding the mechanism by which COVID-19 vaccination and infection cause BTH in PNH patients, regardless of the choice of CI treatment. Bringing awareness to this case of BTH secondary to COVID-19 in a PNH patient treated with pegcetacoplan can further warrant the investigation of the role of COVID-19 in complement disruption and its role in BTH.

## Introduction

Paroxysmal nocturnal hemoglobinuria (PNH) can be a life-threatening hematopoietic stem cell disease and is caused by an acquired defect in the synthesis of the glycosylphosphatidylinositol (GPI) associated proteins CD55 and CD59 [1]. Deficiencies in these proteins lead to complement-mediated red blood cell hemolysis, impaired bone marrow function, and thrombosis. The gold standard for a serologic diagnosis of PNH consists of a flow cytometric analysis to detect antibodies directed against the cell surface markers CD55 and CD59 [2].

Eculizumab is a monoclonal antibody that inhibits complement component 5 (C5) in forming the terminal complement system. Until the advent and clinical use of eculizumab in 2007, the median survival for newly diagnosed PNH patients was 15 to 20 years [3]. Complement inhibitors (CI) have revolutionized treatment modalities for PNH patients resulting in decreased rates of thrombosis, renal failure, and overall mortality [4]. Two other CI have been approved by the U.S. Food and Drug Administration (FDA) for use in PNH treatment: ravulizumab in 2018, a C5 CI, and pegcetacoplan in 2021, an inhibitor of the C3 complement component [5].

This case report discusses the presentation and management of a 29-year-old female with a history of PNH in remission, who was treated with pegcetacoplan, who developed a series of acute hemolytic episodes that were temporally associated with receiving two COVID-19 vaccinations and then a de novo COVID-19 infection. Reports of PNH relapse secondary to COVID-19 vaccination have only been reported in patients receiving eculizumab and ravulizumab. Our search yielded no results for a similar situation in post-COVID-19 vaccinated PNH patients maintained on pegcetacoplan.

## Case presentation

**Figure 1:**
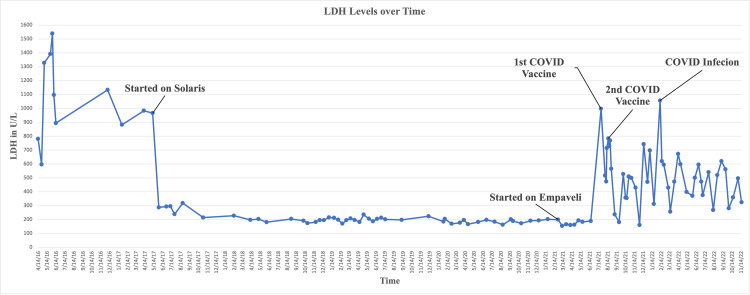
Patient's LDH levels over time with documented treatment adjustments, COVID-19 vaccine dates, and COVID-19 infection dates. Once the patient was started on eculizumab in 2017, there was noticeable serologic remission, with her lactate dehydrogenase (LDH) levels stabilizing in the 200s U/L. Secondary to the patient not achieving symptomatic remission, she was put on pegcetacoplan treatment in February 2021. She then achieved serologic and symptomatic remission for six months until she received her first COVID-19 vaccination in July 2021. Since then, there has been a noticeable serologic relapse as well as moderate symptomatic anemia for the patient.

This patient is a 29-year-old female who was referred to the hematology clinic in 2013 while pregnant at age 20. At that time, she was noted to have a platelet count of 23,000 and was diagnosed with immune thrombocytopenic purpura (ITP). In the subsequent years, the patient was unresponsive to steroid and intravenous immunoglobin (IVIG) therapy. Her diagnosis was modified to PNH in 2017. Eculizumab therapy was initiated in 2017, resulting in complete hematologic remission. In February of 2021, maintenance of eculizumab was no longer effective as the patient developed symptomatic disease activity characterized by severe, worsening fatigue, brain fog, chest pain, and active hemolysis. Under an expanded access protocol, authorization to treat with pegcetacoplan was approved. Treatment commenced in February 2021, resulting in complete clinical and serologic remission (Figure 1).

In July 2021, the patient received her first dose of the Pfizer SARS-CoV-2 vaccine. She subsequently developed fatigue, lethargy, and evidence of active hemolysis. Her condition improved to near remission level with a resolution of fatigue, lethargy, and hemolysis approximately one week after the first vaccination. After receiving her second dose of the Pfizer SARS-CoV-2 vaccine in August of 2021, similar symptoms developed, accompanied by active hemolysis (lactate dehydrogenase (LDH) 783 U/L and a drop in hemoglobin to 7.6 gm/dL).

Subsequent to her receiving the second Pfizer SARS-CoV-2 vaccine, the symptoms outlined above slowly improved but nevertheless persisted, accompanied by continued low-grade hemolysis. She tested seropositive for COVID-19 in January 2022. At that point, she was short of breath (SOB), experiencing exertional chest pain, bleeding from her gums upon brushing her teeth, and easy bruising. Her LDH was 1056 U/L, and her hemoglobin was 6.1 g/d. Platelet and RBC transfusions were administered over two weeks.

Her LDH and hemoglobin have yet to fully stabilize to previous baseline levels since the completion of her Pfizer SARS-CoV-2 vaccination schedule. As of May 2022, the patient requires packed RBC transfusions every two to three months and has undergone a bone marrow transplant evaluation. The patient did not retest following initial seropositivity testing for COVID-19; however, any symptoms directly related to COVID-19 infection have resolved.

## Discussion

This report describes a case of acute hemolysis temporally associated with COVID-19 vaccinations and COVID-19 infection in a patient with previously remitted PNH maintained on pegcetacoplan. Current literature supports a correlation between COVID-19 vaccination and hemolytic episodes in PNH patients treated with older therapies (eculizumab and ravulizumab). However, this is the first report of acute hemolysis associated with COVID-19 vaccinations and acute COVID-19 infection in a patient maintained on pegcetacoplan. Yu et al. report that the spike protein of SARS-CoV-2 competes with complement factor H for heparan sulfate binding and amplifies the alternative complement pathway on the cell surface [6]. However, this conclusion is disputed in recent data, as Gerber et al. suggest that breakthrough hemolysis (BTH) in PNH after the SARS-CoV-2 vaccination is not due to the direct effect of the spike protein. Instead, the authors theorize that a post-vaccination inflammatory response leads to increased complement activity, manifesting clinically as hemolysis [7]. Specifically, they focused on the complement-amplifying conditions that lead to BTH.

Both conclusions may play a role in the cases of BTH reported in the current literature; however, the literature is evident in that both eculizumab and ravulizumab are not protective of these episodes [7,8]. Similarly, our case study suggests that even the newest CI therapy, pegcetacoplan, may leave PNH patients vulnerable and unprotected from BTH after receiving the COVID-19 vaccine. Even with the knowledge of the current literature, there still remains a consensus that the benefits of COVID-19 vaccinations in patients with PNH outweigh the risks [9,10].

## Conclusions

Our case study suggests that the traditional C5 CI (eculizumab, ravulizumab) and the upstream C3 CI (pegcetacoplan) may leave PNH patients vulnerable to BTH after COVID-19 vaccination and infection. However, conflicting reports exist regarding the mechanism by which both COVID-19 vaccination and infection may cause BTH in PNH patients, regardless of the choice of treatment. Thus, the role of COVID-19 in complement disruption and its effects on CI deserves further investigation. This data provides a basis for additional investigation to clarify the mechanism of interaction between the COVID-19 vaccine, the COVID-19 virus, and current PNH CI therapies.
